# LY395756 promotes NR2B expression via activation of AKT/CREB signaling in the juvenile methylazoxymethanol mice model of schizophrenia

**DOI:** 10.1002/brb3.2466

**Published:** 2022-01-13

**Authors:** Meng‐lin Li, Yuan Peng, Ying An, Guo‐yan Li, Yue Lan

**Affiliations:** ^1^ Department of Rehabilitation Guangzhou First People's Hospital School of Medicine South China University of Technology Guangzhou China

**Keywords:** CREB, LY395756, NR2B, schizophrenia

## Abstract

**Introduction:**

Synaptic N‐methyl‐d‐aspartate receptor subtype 2B(NR2B) is significantly reduced in prefrontal cortex (PFC) in the neurodevelopmental methylazoxymethanol (MAM) model of schizophrenia (SCZ). Recent research has shown that LY395756 can effectively restore NR2B levels and improve cognitive performance in juvenile MAM mice model. However, the underlying mechanisms of these beneficial effects remain unclear.

**Materials and methods:**

Juvenile MAM mice model of SCZ is used in our study. Synaptic membrane protein levels were examined by western blotting under different treatment conditions. Interaction of cAMP‐response element binding protein (CREB) and the promoter of NR2B was detected by the chromatin immunoprecipitation (ChIP) assay. Further examination of signaling pathway that mediates NR2B expression was also investigated by western blotting.

**Results:**

In the PFC of the juvenile MAM mice schizophrenia model, CREB was found to directly bind with the promoter of NR2B. LY395756 activated the phosphorylation of AKT. Phosphorylated AKT subsequently induced the phosphorylation of CREB, and the activated CREB promoted the expression of NR2B. Subsequent experiments showed that the dephosphorylation of CREB induced by protein phosphatase 1 (PP1) can inhibit NR2B levels. Taken together, these findings support that the AKT/CREB signaling pathway is essential for the promoting effect of LY395756 on synaptic NR2B in PFC in juvenile MAM mice SCZ model.

**Conclusions:**

Our investigation has identified a novel mechanism by which LY395756 increases NR2B expression in juvenile MAM mice SCZ model. The AKT/CREB signaling pathway warrants further research as a potential direction for clinical treatment of SCZ.

## Graphical abstract

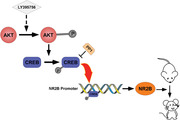

## 1 INTRODUCTION

Schizophrenia (SCZ) is a neurodevelopmental disease with an incidence rate of 0.3%–0.7% worldwide. The symptoms of SCZ develop gradually, and can be categorized as positive, negative, and cognitive symptoms. Symptoms typically begin in young adulthood, with cognitive impairment being the most constantly found symptom and often arising first—sometimes already present in childhood or early adolescence (Insel, 2010; van Os & Kapur, 2009). In recent years, N‐methyl‐d‐aspartate receptor (NMDAR) hypofunction has been reported to be implicated in the molecular mechanisms of SCZ (Law & Deakin, 2001; Meador‐Woodruff & Healy, 2000; Snyder et al., 2013). There is increasing evidence of NMDAR dysregulation—mostly hyporegulation—in postmortem SCZ brains (Beneyto & Meador‐Woodruff, 2008; Martucci et al., 2006; Meador‐Woodruff & Healy, 2000). NMDARs are a family of glutamate receptors. They are heterotetrameric complexes composed of the essential NR1 subunit and other two subunits of either NR2A/D or NR3A/B (Paoletti et al., 2013). It has been documented that the expression of synaptic N‐methyl‐d‐aspartate receptor subtype 2B(NR2B), a subunit of NMDAR, is significantly reduced in the neurodevelopmental methylazoxymethanol (MAM) model of SCZ, and that the function of NMDAR is subsequently weakened (Gulchina et al., 2017; Meltzer et al., 2013; Snyder et al., 2013). Treatments that can effectively enhance NR2B expression and function are thus regarded as promising therapeutic interventions for SCZ.

However, the currently available treatments have low effectiveness. Antipsychotic drugs almost always target dopaminergic and serotoninergic systems rather than glutamatergic receptors (Howes & Kapur, 2009; Miyamoto et al., 2005). As a result, using these drugs to treat cognitive impairment has limited efficacy and is accompanied by strong side effects (Gomes et al., 2016; Miyamoto et al., 2005). Increasing evidence suggests that the metabotropic glutamate receptor 2/3 (mGluR2/3) is related to psychiatric disorders, and that drugs targeting mGluR2/3 provide promising treatment effects and fewer side effects for SCZ patients (Conn et al., 2009; Mezler et al., 2010; Moghaddam & Adams, 1998). Specifically, LY395756, a complex that serves as an mGluR2 agonist/mGluR3 antagonist, has been shown to effectively enhance NMDAR expression and function in normal adult rat prefrontal cortex (M. L. Li, Yang, et al., 2015). Further studies have demonstrated that gestational MAM exposure significantly suppresses NR2B expression, and that treatment with LY395756 can effectively restore NMDAR levels in juvenile MAM‐exposed rats. Furthermore, LY395756 has been shown to alleviate learning deficits and cognitive impairments in juvenile MAM model rats, as determined by performance on cross‐maze based set‐shifting task (M. L. Li et al., 2017). These findings suggest that activation of metabotropic glutamate receptors may be a promising treatment to enhance NMDAR function and ameliorate cognitive impairment in SCZ. However, the underlying mechanisms through which LY395756 reverses cognitive impairment in MAM‐SCZ model animals remain unclear.

The cAMP‐response element binding protein (CREB) plays an important role in central nervous system neurons. CREB regulates the expression of genes and serves as the substrate for AKT and GSK3β phosphorylation (Ren et al., 2014). There is emerging evidence that CREB is involved in the pathological mechanisms of several neurological disorders (Mohammadi et al., 2018), including SCZ (Einoch et al., 2017; X. Y. Li et al., 2011). Several studies have reported a relationship between NMDAR and CREB. Activation of NMDAR (composed of NR2B > NR2A) can dephosphorylate CREB at ser133 and lead to neuronal death (Rittase et al., 2014); therefore, we hypothesize that there is a relationship between CREB and NMDAR. In our study, we found that in the juvenile MAM mice SCZ model, CREB can directly bind with NR2B promoter and LY395657 restores the expression of NR2B through the AKT/CREB signaling pathway. Our data will provide novel sight for the treatment of SCZ in the future.

## 2 MATERIALS AND METHODS

### 2.1 Animals and treatments

Pregnant C57/BL6J mice at embryonic day 15 (E15) were purchased from the Beijing Vital River Laboratory Animal Technology Co., Ltd (Beijing, China). Animals were raised in temperature‐controlled housing (22 ± 2°C) with a 12‐h light/dark cycle, 45 ± 15% humidity and had access to food and water ad libitum. All animal experiments were approved by the Guangzhou First People's Hospital Animal Care and Use Committee and carried out according to the National Institutes of Health Guide for the Care and Use of Laboratory Animals guidelines. Pregnant mice at E15 to E17 were injected once daily with either neurotoxin MAM (10 mg/kg, i.p.) for 3 days to establish the SCZ animal model or 0.9% physiological saline (1 ml/kg) as a vehicle control. When pups grew up to postnatal day 10 (P10), all male offspring were chosen and weaned at P21 and rehoused in groups of 4–6 mice. Only the P21 juvenile male pups were included in the study.

To examine NMDAR dysregulation of juvenile MAM‐SCZ animal model and the effects of LY39 on NMDAR expression, western blotting was applied for the detection of synaptic membrane protein from prefrontal cortex (PFC). LY39 at a dose of 0.3, 1.0, or 3.0 mg/kg (single dose, intraperitoneally, i.p.) was administered 1 h before the mice were sacrificed for tissue collection. LY39 usually peaks within 30 min of brain concentration after treatment. Therefore, we collected synaptic membrane protein from PFC after 1 h of LY39 administration. Animals were divided into five groups as follows: the saline‐saline group, the MAM‐saline group, the MAM‐LY0.3 group, the MAM‐LY1.0 group, and the MAM‐LY3.0 group. In additional experiments, the CREB inactivator protein phosphatase 1 (PP1) (25 mg/kg, single dose, i.p.) was administered simultaneously with LY39 on juvenile P21 MAM mice. PP1 also reached a maximum level 30 min after the injection. Synaptic membrane proteins from PFC were collected after 1 h of LY39 and PP1 administration. Each group included at least eight animals, and samples from each animal were run at least four times.

Primary PFC neurons were obtained from P21 male MAM mice. According to the previous described methods (Wang et al., 2013), PFC tissues were removed in cold dissection medium (10 mM HEPES, 33.3 mM glucose, 12 mM MgSO_4_, 5 μg/ml gentamicin, and 0.3% bovine serum albumin [BSA] in Hank's buffered salt solution [HBSS]) and dissociated with trypsin at 37°C. Then, we used cell filter to treat neurons and rinsed with neurobasal media (which contains 0.5% glutamine, 100 μg/ml penicillin, and 100 μg/ml streptomycin) supplemented with B27 and 5% fetal bovine serum (FBS). At 37°C with 5% CO_2_, on 6‐well plates coated with poly‐d‐lysine, neurons were then plated in growth medium (neurobasal media plus 2% B27 supplement and 5% FBS) at a density of 100,000 cells/cm^2^ for 4 h. Afterward, cells were maintained in neurobasal medium supplemented with B27 and l‐glutamine without FBS at 37°C/5% CO_2_. Cells were fed two times per week and half of the media was changed each time. Primary PFC MAM neurons were prepared for chromatin immunoprecipitation (ChIP) assay.

LY395756 (LY39) and PP1 were purchased from Tocris Bioscience (Minneapolis, MN, USA), and MAM acetate was purchased from MRIGlobal Chemical Carcinogen Repository (Kansas City, MO, USA). All animals were deeply anesthetized with euthasol (0.2 ml/kg, Virbac Animal Health).

### 2.2 Synaptic membrane protein collection

Following our laboratory's previously described protocol (M. L. Li et al., 2017), P21 male mice were decapitated and the forebrain containing PFC was quickly dissected and homogenized using cold lysis buffer (in mM: 320 sucrose, 4 HEPES‐NaOH buffer, pH 7.4, 2 EGTA, 1 sodium orthovanadate, 0.1 phenylmethylsulfonyl fluoride, 10 sodium fluoride, 10 sodium pyrophosphate, with 1 μg/ml leupeptin and 1 μg/ml aprotinin). The lysates were then centrifuged at 1,000 *g* for 10 min at 4°C. The supernatant was collected and centrifuged again at 15,000 *g* for 15 min at 4°C. The pellet was resuspended in lysis buffer and centrifuged at 15,000 *g* for an additional 15 min at 4°C to produce synaptosomes. The synaptosomal fraction was then hypoosmotically lysed and centrifuged at 25,000 *g* for 30 min at 4°C to collect the synaptosomal plasma membranes. Lysis buffer was added to the pellet to produce the final samples. These samples were stored at −80°C for future use or an aliquot was made and stored at −20°C for immediate use.

### 2.3 Western blot assay

In order to determine protein concentration before the western blotting, a bicinchoninic acid (BCA) protein assay was performed. Next, equal protein samples were prepared with 4× laemmli and lysis buffer and boiled for 5 min. Samples were isolated on a 7.5% SDS‐PAGE gel and transferred to a polyvinylidene fluoride (PVDF) membrane (Millipore). The membrane was probed using primary antibody overnight at 4°C. Each blot was used to probe multiple antibodies, including anti‐rabbit NR2B (Abcam, ab254356, 1:1000), anti‐rabbit pNR2B‐Tyr1472 (ThermoFisher Scientific, 38–7000, 1:1000), anti‐rabbit p‐CREB (Abcam, ab32096, 1:5000), anti‐rabbit p‐AKT (Abcam, ab8805, 1:1500), anti‐rabbit p‐KAC (Cell Signaling Technology, 4781, 1:1000), anti‐mouse p‐CAMKII (Abcam, ab171095, 1:2000), anti‐rabbit p‐ERK (Abcam, ab229912, 1:1000), anti‐rabbit p‐MSK1 (Abcam, ab278550, 1:5000), and anti‐mouse actin (Sigma, A5316, 1:100,000) served as a loading control. The membrane was then incubated with horseradish peroxidase (HRP)‐conjugated anti‐rabbit or anti‐mouse IgG secondary antibody for 1 h at room temperature. Lastly, the signal was detected using the Super Signal Chemi‐luminescent Substrate (Pierce).

### 2.4 ChIP assay

ChIP assays were performed in primary PFC neurons of P21 juvenile MAM mice treated with LY37 at 100 μM or with culture medium as vehicle control for 1 h, and each group contains 10^7^ cells. The next step is to cross‐link cells by adding directly to the medium formaldehyde to a final concentration of 1% at room temperature. Ten minutes later, we added ice‐cold PBS placed the plates on ice, washed with PBS, and scraped the cells. After centrifugation, cells were lysed in lysis buffer (50 mM Tris at pH 8.0, 5 mM EDTA, 1% SDS) supplemented with proteases inhibitors. Chromatin was sheared by sonication to produce DNA fragments (150 and 200 bp in size). Cells were centrifuged again and took out 5% of the sample as INPUT, diluted 10 times with dilution buffer (50 mM Tris at pH 8.0, 0.5% Triton X‐100, 0.1 M NaCl, 2 mM EDTA) supplemented with protease inhibitors. Extracts were precleared for 2 h at 4°C using 2 mg of sheared salmon sperm DNA (Invitrogen, Inc.) and 45 ml of protein A‐Sepharose (50% slurry in dilution buffer). Immunoprecipitations were carried out overnight at 4°C with 2 μg of CREB anti‐rabbit antibody. Meanwhile, supernatants were incubated with normal rabbit serum as controls. Immune complexes were collected with protein A‐Sepharose and washed three times (5 min each) with low salt buffer (20 mM Tris at pH 8.0, 0.1% SDS, 1% Triton, 2 mM EDTA, 200 mM NaCl) and three times with TE (Tris‐EDTA buffer). Immune complexes were extracted with 1% SDS (v/v), 0.1 M NaHCO3, and heated overnight at 65°C to reverse the cross‐linking. After proteinase K digestion (100 mg, 1 h at 50°C), DNA fragments were purified on QIAquick Spin columns (Qiagen) in 50 ml of elution buffer (EB) and 1 ml was used in each QPCR. The primers for amplification of the NR2B promoter are as follows:

P1, forward, 5′‐TAGAGTACTGGTGTTGTGG‐3′, reverse, 5′‐ ATTTTAAAACAACAATTCTTTGAT‐3′;
P2, forward, 5′‐GCCAGAAAAAAAAA TTGTGT‐3′, reverse, 5′‐TTGCAGAGTTCTAAAATGCTC‐3′;
P3, forward, 5′‐GATA GCCATTTTGCAAGAGTG‐3′, reverse, 5′‐ CCCAGAACTAAAGCTCCGAT‐3′;
P4, forward, 5′‐TGAATAAAATGCTATTTAACTGGAAAAT‐3′, reverse, 5′‐ AAAAC AAAATTTACGCTAAACCAAT‐3′; and
P5, forward, 5′‐AATCTTGTGTCTTGAAGAG AT‐3′, reverse, 5′‐TTTCCCACCTCTCATCCGT −3′.

The ChIP assay was implemented using the Immunoprecipitation Assay Kit (Millipore). DNA from ChIP was quantified by qPCR using SYBER Green incorporation (Applied Biosystems). PCR (SYBER Green) analysis was performed on an Applied Biosystems 7300 real‐time PCR system.

### 2.5 Statistical analysis

All data are presented as mean ± standard error. Data between two groups were compared using the unpaired Student's *t*‐test (with equal variances) or *F*‐test (with unequal variances). Comparisons of multiple groups were carried out with analysis of variance (ANOVA) with SPSS followed by Tukey's post hoc test to compare between individual groups. All statistical analyses were performed using SPSS 22.0 software, and a *p*‐value < .05 was considered statistically significant.

## 3 RESULTS

### 3.1 Prenatal injection of MAM induces NR2B downregulation, and LY395756 restores NR2B level in juvenile MAM‐exposed mice PFC

**FIGURE 1:**
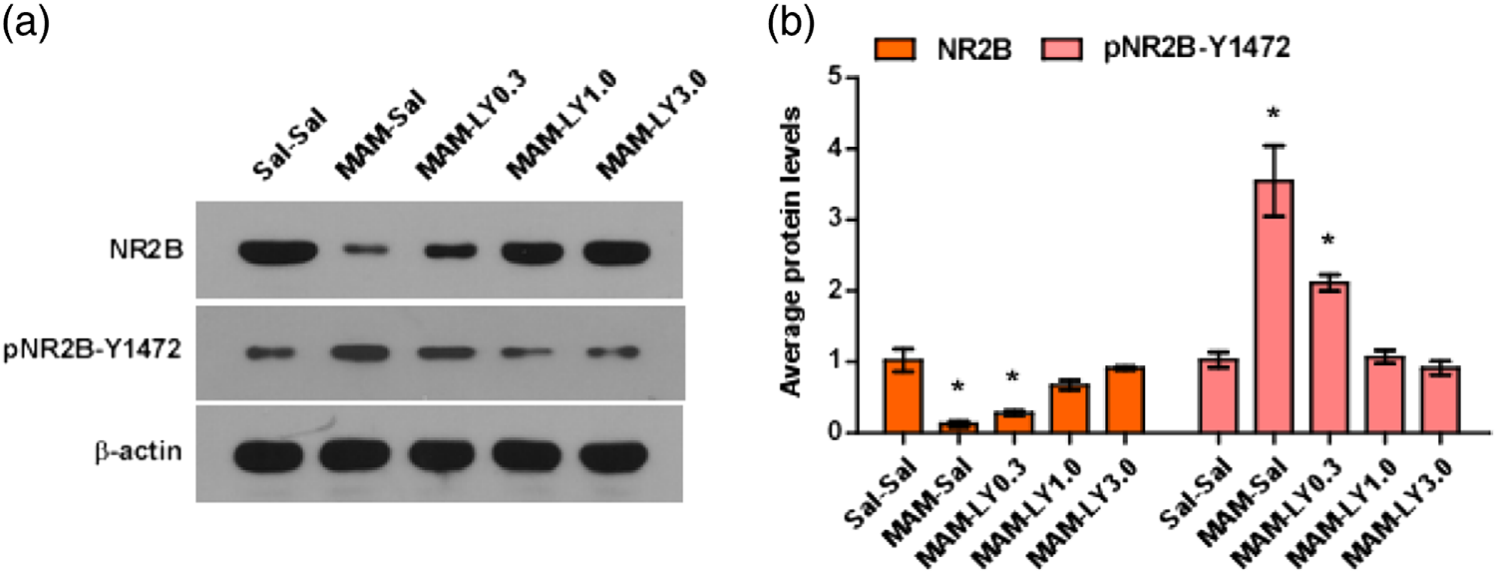
Prenatal injection of methylazoxymethanol (MAM) (10 mg/kg, i.p.) induced N‐methyl‐d‐aspartate receptor (NMDAR) dysregulation in the juvenile mice prefrontal cortex (PFC) and in vivo treatment with LY395756 significantly reverses the downregulation of N‐methyl‐d‐aspartate receptor subtype 2B (NR2B) in PFC in P21 juvenile MAM mice to a normal level compared with the saline‐saline control. (a) Representative western blots of the saline‐saline control, saline and LY395756 administration at 0.3, 1.0, or 3.0 mg/kg in juvenile MAM mice. (b) Summary histogram shows the synaptic membrane protein NR2B and phosphorylation Y1472 of NR2B in PFC from P21 juvenile saline‐ and MAM‐exposed animals. There was a significant decrease in NR2B and a significant increase in p‐NR2B‐Y1472 in MAM‐exposed mice compared with saline‐saline control mice, and 0.3, 1.0, or 3.0 mg/kg LY39 all successfully reverses the dysregulation of NR2B and p‐NR2B‐Y1472 to a normal level. *p < .05, **p < .01 compared with saline‐saline control

First, we detected the synaptic protein expression of NR2B and its major phosphorylation site, pNR2BTyr1472 (Y1472), which is reported to be implicated in NMDAR synaptic insertion (Chen & Roche, 2007; Hallett et al., 2006; Petralia et al., 2009). The western blot results of synaptic membrane protein from PFC showed that there was a significant decrease in NR2B protein level and a significant increase in Y1472 when compared from P21 juvenile MAM mice treated with saline (MAM‐Sal) with P21 Sal mice treated with saline (Sal‐Sal) (*n* = 6 mice for both MAM‐Sal and Sal‐Sal group, *p* = .0054 for NR2B and *p* = .0078 for Y1472; Figure 1). After LY39 administration, synaptic protein NR2B expression in PFC was successfully reversed to the normal level (compared with juvenile Sal‐Sal mice) in a dose‐dependent manner relative to saline treatment in P21 juvenile MAM‐exposed mice (Figure 1, MAM‐Sal vs. MAM‐LY0.3, *p* = .0391; vs. MAM‐LY1.0, *p* = .0021; vs. MAM‐LY3.0, *p* < .001; Sal‐Sal vs. MAM‐LY0.3, *p* = .0102; vs. MAM‐LY1.0, *p* > .05; vs. MAM‐LY3.0, *p* > .05). Treatment of LY39 at 3.0 mg/kg had a significant stronger effect compared with 0.3 mg/kg (*p* < .001) and 1.0 mg/kg (*p* = .0276). Likewise, the phosphorylation level of Y1472 dramatically decreased to the normal level (compared with juvenile Sal‐Sal mice) in a dose‐dependent manner in MAM‐exposed mice treated with LY39 relative to saline treatment in P21 juvenile MAM mice (Figure 1, MAM‐Sal vs. MAM‐LY0.3, *p* = .0458; vs. MAM‐LY1.0, *p* = .0081; vs. MAM‐LY3.0, *p* = .0066; Sal‐Sal vs. MAM‐LY0.3, *p* = .0022; vs. MAM‐LY1.0, *p* > .05; vs. MAM‐LY3.0, *p* > .05). Treatment of LY39 at 3.0 mg/kg had a significant stronger effect compared with 0.3 mg/kg (*p* = .0013) and 1.0 mg/kg (*p* = .0432). These data suggest that MAM exposure at juvenile period induced an NMDAR misexpression, which presented as NR2B downregulation and Y1472 upregulation in the PFC and LY39 is effective in reversing juvenile NMDAR dysregulation in the PFC of MAM‐exposed mice.

### 3.2 CREB directly binds with the P5 region of NR2B promoter

**FIGURE 2:**
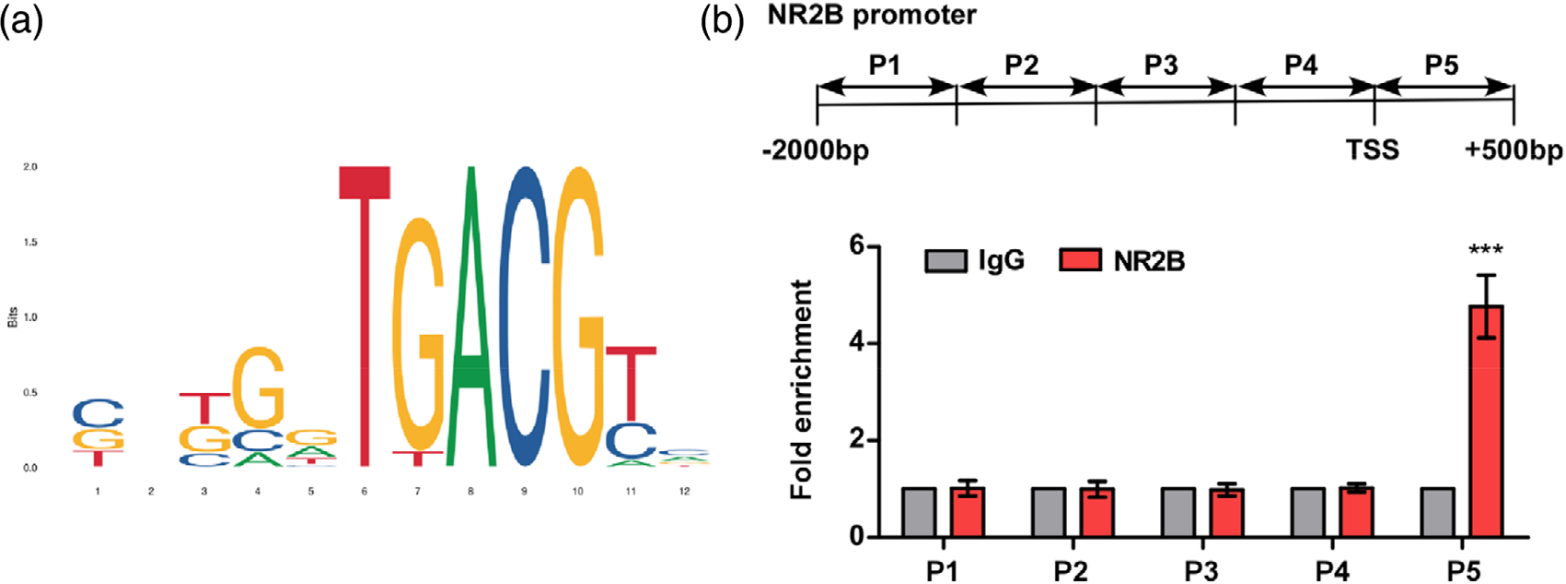
cAMP‐response element binding protein (CREB) can directly bind with the P5 region of the N‐methyl‐d‐aspartate receptor subtype 2B (NR2B) promoter. (a) Based on transcription factor binding profile database, illustration of the potential response elements of NR2B promoter that CREB may interact with. (b) chromatin immunoprecipitation (ChIP) assay showed that CREB directly binds with the P5 region of NR2B promoter. ChIP analysis for binding NR2B promoter to CREB antibody or control IgG. Further qPCR demonstrates CREB activates transcriptional regulation of NR2B by directly binds with P5 region. ***p < .001 versus IgG

We then investigated the mechanisms by which LY39 restored synaptic NR2B and NR2B‐Y1472 expression in PFC in juvenile MAM mice. Promoter analysis identified that CREB might be as a transcription factor regulating the expression of NR2B directly, as shown by the public the Transcriptional Regulatory Element Database (TRED, http://rulai.cshl.edu/TRED) and JASPAR (http://jaspar.genereg.net/) databases (Figure 2a). The regions of the NR2B promoter (defined as response elements) that CREB can interact with are illustrated in Figure 2a.

To confirm this prediction, the ChIP assay was performed and different regions of the NR2B promoter were amplified with qPCR experiment. As illustrated in Figure 2b, the results showed that CREB can directly bind with the P5 region of the NR2B promoter (for P5 region, *p* = .0043; for other regions, *p* > .05), suggesting CREB is involved in the transcriptional regulation of NR2B protein synthesis in PFC in juvenile MAM mice.

### 3.3 Treatment of LY39 may regulate NR2B expression through AKT/CREB signaling pathway in juvenile MAM mice PFC

Based on the ChIP assay, we hypothesized that LY39 may regulate PFC synaptic NR2B expression in juvenile MAM mice through inducing the transcriptional activity of CREB. Previous studies indicated that the transcriptional activity of CREB is activated at serine residues—especially serine 133 and serine 142—through various kinases, including PKA, CaMK, Ras/MAPK, MSK1, and AKT (Niwano et al., 2018). Phosphorylation at ser133 is known to stimulate the transcriptional activation of CREB, while phosphorylation at ser142 induces dissociation of the CREB dimer and reduces the transcriptional activation of CREB (Carlezon et al., 2005). Therefore, we evaluated these signaling kinases proteins after 1 h of LY39 administration to detect the signaling pathways which LY39 affect the phosphorylation level of CREB‐ser133.

**FIGURE 3:**
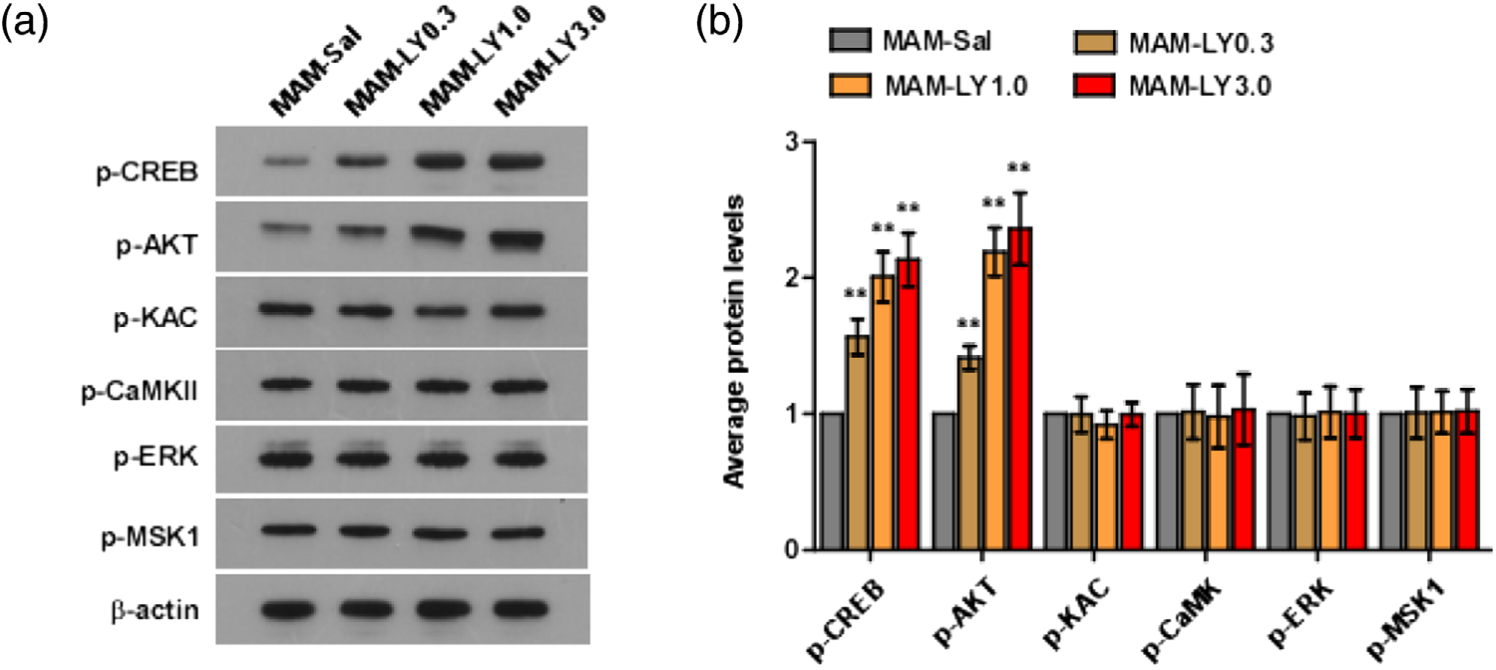
Treatment with LY395756 significantly increases the synaptic membrane phosphorylation protein levels of AKT and cAMP‐response element binding protein (CREB) in a dose‐dependent manner in the juvenile methylazoxymethanol (MAM) mice prefrontal cortex (PFC), suggesting that LY39 might affect the p‐AKT/p‐CREB signaling axis in the schizophrenia animal model. (a) Representative western blots of p‐CREB, p‐AKT, p‐KAC, p‐CaMKII, p‐ERK, and p‐MSK1 in juvenile MAM mice treated with saline or LY395756 of 0.3, 1.0, or 3.0 mg/kg. (b) Summary histograms of the synaptic membrane protein of p‐CREB, p‐AKT, p‐KAC, p‐CaMKII, p‐ERK, and p‐MSK1 expressions in juvenile MAM mice PFC. There was a significant increase in p‐CREB and p‐AKT when treated with LY395756 at 0.3, 1.0, or 3.0 mg/kg in juvenile MAM mice compared with juvenile MAM mice treated with saline control. **p < .01 compared with MAM‐saline control

The western blot results (Figure 3) showed that the phosphorylation level of CREB (ser133) was significantly increased in a dose‐dependent manner in juvenile MAM‐exposed mice treated with LY39 at 0.3, 1.0, and 3.0 mg/kg relative to MAM‐SAL control (*n* = 8 mice for each group, for p‐CREB‐ser133, MAM‐SAL vs. MAM‐LY0.3, *p* = .0120; vs. MAM‐LY1.0, *p* = .0055; vs. MAM‐LY3.0, *p* = .0045). Similarly, the level of p‐AKT was significantly increased in a dose‐dependent manner after LY39 treatment at 0.3, 1.0, and 3.0 mg/kg compared with MAM‐SAL control (*n* = 8 mice for each group, for p‐AKT, MAM‐SAL vs. MAM‐LY0.3, *p* = .0085; vs. MAM‐LY1.0, *p* = .0025; vs. MAM‐LY3.0, *p* = .0069). However, the levels of p‐KAC, p‐CaMKII, p‐ERK, and p‐MSK1 were not significantly changed by LY39 treatment at 0.3, 1.0, and 3.0 mg/kg relative to MAM‐SAL control (*n* = 8 mice for each group, *p* > .05 for all groups). Taken together, these data suggest that administration of LY39 may regulate synaptic NR2B expression in PFC in juvenile MAM mice through ser133 phosphorylation site to activate CREB transcriptional activity inducing by AKT in a dose‐dependent manner, but not through PKA, CaMK, Ras/MAPK, or MSK1 activating on CREB, suggesting that LY39 might restore synaptic NMDAR function in PFC by reversing NR2B expression in juvenile MAM mice via AKT/CREB signaling pathway.

### 3.4 Inactivation of CREB inhibits the promoting effect of LY39 on synaptic NR2B expression in PFC in juvenile MAM mice

**FIGURE 4:**
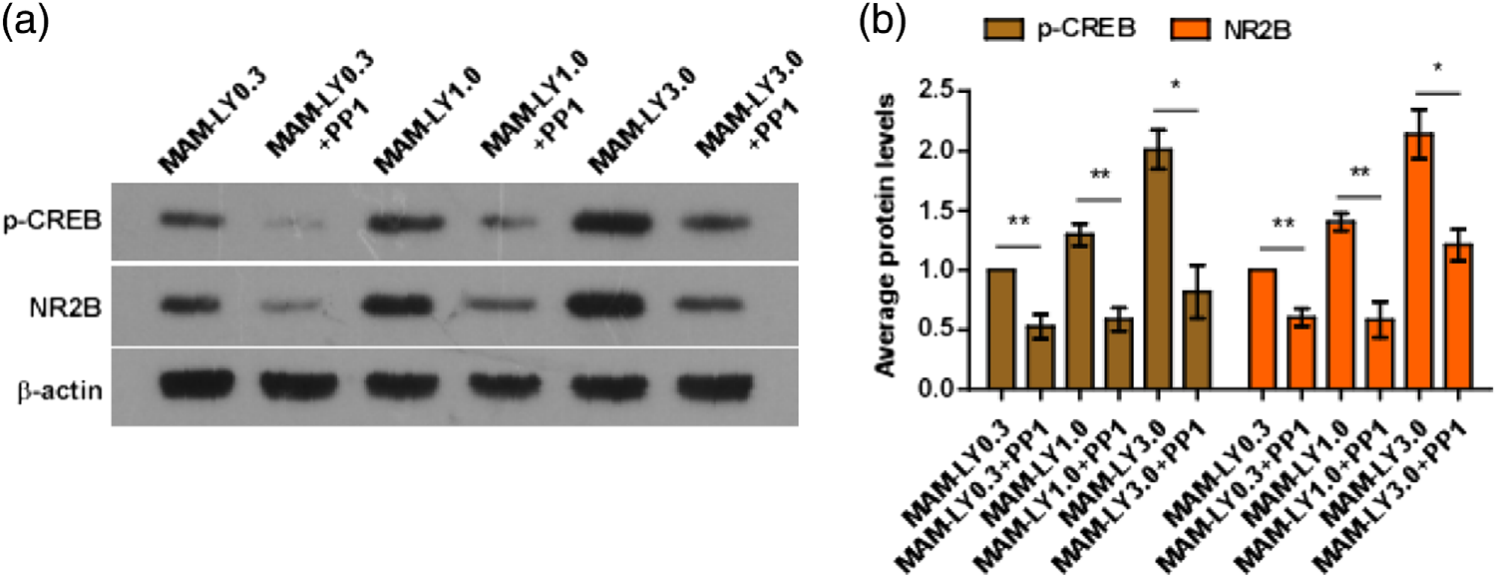
The upregulation of synaptic membrane protein N‐methyl‐d‐aspartate receptor subtype 2B (NR2B) induced by LY395756 in juvenile methylazoxymethanol (MAM) mice prefrontal cortex (PFC) was inhibited by inactivation of cAMP‐response element binding protein (CREB) by protein phosphatase 1 (PP1), confirming that LY39 may regulate NR2B in PFC in MAM animal model through AKT/CREB signaling pathway. (a) Representative western blots of synaptic membrane p‐CREB and NR2B in PFC in juvenile MAM mice treated with LY39 only or LY39+PP1. (b) Summary histograms of the synaptic membrane protein of p‐CREB and NR2B in juvenile MAM mice PFC treated with LY39 only or LY39+PP1. There was a significant decrease in p‐CREB after treated with PP1, suggesting the inactive effect of PP1 on CREB Further results showed PP1 significantly decreased NR2B synaptic membrane protein level reversed by LY39 treatment at all 0.3, 1.0, 3.0 mg/kg in juvenile MAM mice PFC when compared with the juvenile MAM mice treated with only LY39 as controls. *p < .05, **p < .01 compared with juvenile MAM mice treated with only LY39 at 0.3, 1.0, and 3.0 mg/kg, respectively, as controls

Based on the previous experiments, protein levels of p‐AKT and p‐CREB‐ser were significantly increased with LY39 treatment. We therefore further determined whether AKT/CREB signaling pathway is involved in regulating synaptic NR2B expression in PFC in juvenile MAM mice. AKT/CREB signaling pathway has been reported to be involved in maintaining normal function of the nervous system, including regulating synaptic plasticity of neurons, modulating growth and proliferation of neurocytes (Hu et al., 2017; Qu et al., 2016). Previous studies reported that PP1 plays a critical role in synaptic plasticity and memory, and brings out a negative regulative effect on CREB that leads to dephosphorylation and deactivation (Genoux et al., 2002; Peters et al., 2009). We therefore used CREB inactivator PP1 (25 mg/kg, i.p.) to deactivate CREB phosphorylated at ser133, and assess whether increase of NR2B expression induced by LY39 was inhibited. PP1 was administered simultaneously with LY39 or saline injection 1 h before juvenile MAM mice were sacrificed for collecting synaptic membrane proteins from PFC tissues. As illustrated in Figure 4, PP1 effectively and significantly inhibited p‐CREB expression (*n* = 8 for each group, MAM+LY0.3 vs. MAM+LY0.3+PP1, *p* = .0096; MAM+LY1.0 vs. MAM+LY1.0+PP1, *p* = .0059; MAM+LY3.0 vs. MAM+LY3.0+PP1, *p* = .0125). Moreover, the increased expression of NR2B induced by LY39 was also significantly reduced after administration of PP1 (n = 8 for each group, MAM+LY0.3 vs. MAM+LY0.3+PP1, *p* = .0062; MAM+LY1.0 vs. MAM+LY1.0+PP1, *p* = .0081; MAM+LY3.0 vs. MAM+LY3.0+PP1, *p* = .0193). Together, these results support that AKT/CREB signaling pathway plays an important role in the promoting effect of LY39 on synaptic NR2B expression in PFC.

## 4 DISCUSSION

In the present study, we found that CREB can directly bind with the promoter sequences of NR2B. LY395756 increased NR2B levels by activating the AKT/CREB signaling pathway. Subsequent experiments showed that the AKT/CREB signaling pathway is essential for the promoting effect of LY395756 on synaptic NR2B in the PFC in juvenile MAM mice model. Our results uncovered a novel mechanism through which LY395756 increases the levels of NR2B, which might provide new ideas for the clinical treatment of SCZ.

In light of evidence that SCZ begins in adolescence or even childhood, it is possible that current treatments for SCZ miss the optimal time window for intervention. Therefore, giving individuals effective and early treatment is a potential method for helping SCZ patients. NMDAR hypofunction is regarded as one of the main pathological mechanisms in SCZ (Snyder et al., 2013). In the juvenile period, MAM‐exposed rats present with dysregulated NMDAR expression, including a significant decrease in NR2B and a significant increase in pNR2BY1472 (M. L. Li et al., 2017). However, when these animals reach adulthood, NMDAR expression is comparable to that of control animals, yet cognitive impairment remains (M. L. Li, Yang, et al., 2015). Therefore, drugs that target and repair NR2B expression in the juvenile period can be viewed as promising for SCZ. In the last 10 years, mGluR2 agonists have gained increasing attention for their applications in neurological and psychiatric disorders (Engel et al., 2016; Imre et al., 2006; M. L. Li, Hu, et al., 2015), especially for the treatment of SCZ (Conn et al., 2009; Fell et al., 2012; Patil et al., 2007; Xing et al., 2018). Thus, in the present study, we explored the effects of the mGluR2 agonist/mGluR3 antagonist LY395756 on NMDAR expression in saline‐exposed and MAM‐exposed mice, finding that LY395756 successfully increased NR2B and decreased p‐NR2B‐Y1472 expression. This result is consistent with our previous findings in juvenile MAM‐exposed rats (M. L. Li et al., 2017), and again demonstrates the ability of LY395756 to reverse dysregulated NR2B expression in the MAM‐SCZ animal model.

Subsequently, we explored the specific mechanisms through which LY395756 regulates NR2B. Using the ChIP assay, we confirmed that CREB can directly bind with the P5 region of the NR2B promoter. To the best of our knowledge, this is the first report of a direct relationship between an NMDAR, specifically NR2B, and CREB. Huang et al. (2019) noted the possibility that CREB and NR2B play important roles in mental disorders; however, the relationship between CREB and NR2B was not described. Nevertheless, whether direct binding between CREB and NR2B is mediated by other mediums is yet to be explored. CREB was initially identified in 1987. It belongs to the large family of basic leucine zipper (bZIP)‐containing transcription factors that interact with promoter cAMP responsive element (CRE) sites. C‐jun, c‐fos, and c‐myc also belong to this family. CREB is known to modulate multiple biological processes, including cell differentiation and cell growth (Steven & Seliger, 2016). Emerging evidence supports that CREB is involved in learning and memory (Saura & Cardinaux, 2017) and can exert therapeutic effects as the antidepressants in neurological disorders involving the hippocampus (Carlezon et al., 2005). After phosphorylated, CREB at serine 133 activates CREB‐mediated gene transcription and activates the PI3K‐AKT or Ras‐MEK‐ERK pathway. (Du & Montminy, 1998; Finkbeiner, 2001; Johannessen & Moens, 2007; Mayr & Montminy, 2001). Our results showed that LY395756 treatment can increase the phosphorylation levels of CREB at the ser133 phosphorylation site. Further research showed that p‐CREB increases through promotion of the phosphorylation of AKT. In addition, reducing the phosphorylation levels of CREB by PP1 inhibited the expression of NR2B. Together, these results provide further evidence that the AKT/CREB signaling pathway is essential for the promoting effect of LY395756 on NR2B. Some studies found that poor sleep was associated with schizophrenia (Chang et al., 2021).

However, poor sleep quality was common among university students (Liu et al., 2021;). More study should be done in the future.

In conclusion, our results have revealed a new mechanism of AKT/CREB through which LY395756 promotes the expression of synaptic NR2B in PFC in juvenile MAM‐SCZ mice model and demonstrated the important direct binding relationship between CREB and NR2B, thereby successfully reversing NMDAR dysregulation in the MAM‐mice model of SCZ. Our findings may offer novel insights for the clinical application of mGluR2 agonists to treat SCZ, which may have important implications for future clinical interventions.

## CONFLICT OF INTEREST

The authors declare no conflict of interest.

### PEER REVIEW

The peer review history for this article is available at https://publons.com/publon/10.1002/brb3.2466.

## Data Availability

The data that support the findings of this study are available from the corresponding author upon reasonable request.
